# Parametric response mapping of co-registered intravoxel incoherent motion magnetic resonance imaging and positron emission tomography in locally advanced cervical cancer undergoing concurrent chemoradiation therapy

**DOI:** 10.1016/j.phro.2024.100630

**Published:** 2024-08-18

**Authors:** Dante P.I. Capaldi, Jen-Yeu Wang, Lianli Liu, Vipul R. Sheth, Elizabeth A. Kidd, Dimitre H. Hristov

**Affiliations:** a Department of Radiation Oncology, University of California San Francisco, San Francisco, CA, USA; b Department of Radiation Oncology, School of Medicine, Stanford University, Stanford, CA, USA; c Department of Radiology, School of Medicine, Stanford University, Stanford, CA, USA

**Keywords:** Cervical Cancer, Parametric Response Mapping, Intravoxel Incoherent motion, Magnetic Resonance Imaging, Positron Emission Tomography, Treatment Response

## Abstract

**Background and Purpose:**

Intravoxel-incoherent-motion (IVIM) magnetic-resonance-imaging (MRI) and positron-emission-tomography (PET) have been investigated independently but not voxel-wise to evaluate tumor microenvironment in cervical carcinoma patients. Whether regionally combined information of IVIM and PET offers additional predictive benefit over each modality independently has not been explored. Here, we investigated parametric-response-mapping (PRM) of co-registered PET and IVIM in cervical cancer patients to identify sub-volumes that may predict tumor shrinkage to concurrent-chemoradiation-therapy (CCRT).

**Materials and Methods:**

Twenty cervical cancer patients (age: 63[41–85]) were retrospectively evaluated. Diffusion-weighted-images (DWIs) were acquired on 3.0 T MRIs using a free-breathing single-shot-spin echo-planar-imaging (EPI) sequence. Pre- and on-treatment (∼after four-weeks of CCRT) MRI and pre-treatment FDG-PET/CT were acquired. IVIM model-fitting on the DWIs was performed using a Bayesian-fitting simplified two-compartment model. Three-dimensional rigidly-registered maps of PET/CT standardized-uptake-value (SUV) and IVIM diffusion-coefficient (*D*) and perfusion-fraction (*f*) were generated. Population-means of PET-SUV, IVIM-*D* and IVIM-*f* from pre-treatment-scans were calculated and used to generate PRM via a voxel-wise joint-histogram-analysis to classify voxels as high/low metabolic-activity and with high/low (hi/lo) cellular-density. Similar PRM maps were generated for SUV and *f*.

**Results:**

Tumor-volume (p < 0.001) significantly decreased, while IVIM-*f* (p = 0.002) and IVIM-*D* (p = 0.03) significantly increased on-treatment. Pre-treatment tumor-volume (r = -0.45,p = 0.04) and PRM-SUV^hi^*D*^lo^ (r = -0.65,p = 0.002) negatively correlated with ΔGTV, while pre-treatment IVIM-*D* (r = 0.64,p = 0.002), PRM-SUV^lo^*f*^hi^ (r = 0.52,p = 0.02), and PRM-SUV^lo^*D*^hi^ (r = 0.74,p < 0.001) positively correlated with ΔGTV.

**Conclusion:**

IVIM and PET was performed on cervical cancer patients undergoing CCRT and we observed that both IVIM-*f* and IVIM-*D* increased during treatment. Additionally, PRM was applied, and sub-volumes were identified that were related to ΔGTV.

## 1 Introduction

Multi-modal imaging has been used to non-invasively interrogate the tumor microenvironment in cervical carcinoma patients [1], [2], [3], [4]. The metabolic activity of the tumor has primarily been imaged using positron emission tomography (PET), while tumor perfusion has been assessed using dynamic contrast enhanced (DCE) imaging, either using computed tomography (CT) or magnetic resonance imaging (MRI). Both ^18^F-fluorodeoxyglucose (FDG) PET/CT and DCE imaging have shown to be independently related to prognosis [5], [6], [7], response to chemoradiation [8], [9], [10], and long-term outcomes [11], [12]. While only weak correlations between PET and DCE CT were observed in a previous study [13], more recently, combining these metrics of the tumor microenvironment via parametric response mapping (PRM), a voxel-wise approach for image analysis and quantification of sub-volumes within co-registered image datasets [14], [15], [16], [17], has shown to better identify radioresistant sub-volume, over PET and DCE CT alone [18]. Although promising, a challenge with DCE is the need for contrast agents (both CT and MRI) as well as additional radiation dose (CT alone). Consequently, this has motivated the use of diffusion weighted imaging (DWI) derived intravoxel incoherent motion (IVIM) MRI [19], which provides both measurements of microcirculatory perfusion and cell density, as an alternative to DCE imaging [20], but does not require contrast.

Several studies have investigated the use of IVIM MRI in locally advanced cervical cancer patients to evaluate treatment outcomes and predict prognosis [21], [22], [23], [24], [25], [26]. Furthermore, a recent study utilizing a hybrid PET/MRI system demonstrated the combined role that PET and IVIM in locally advanced cervical cancer can provide to predict tumor recurrence [25]. Specifically, it was observed that the combined pre-treatment PET, lymph node (LN) status, as well as changes in IVIM diffusion (between pre- and on-treatment timepoints) best identified locally advanced cervical cancer patients with high risk of recurrence post concurrent chemoradiation therapy (CCRT). In a similar fashion, here, our objective was to first investigate changes in IVIM in cervical cancer patients undergoing CCRT and evaluate the relationship between these metrics with changes in gross tumor volume (GTV) and FDG PET/CT. Tumor shrinkage, via changes in GTV, has been shown in prior literature to be related as a surrogate measurement of treatment response (local control rate) in cervical cancer patients receiving CCRT [27], [28]. Additionally, our objective was to investigate PRM analysis of the pre-treatment co-registered PET/CT and IVIM MRI to identify sub-volumes that may predict response to treatment. Accordingly, we hypothesize that greater changes in IVIM MRI would be associated with larger changes in tumor volume for patients receiving CCRT. Furthermore, we hypothesize that the complementary information of the combined IVIM MRI and metabolic activity quantified by FDG-PET/CT imaging via PRM analysis will spatially provide sub-volumes that would be related to change in tumor burden.

## 2 Materials and methods

### 2.1 Study design and patient eligibility

In this IRB approved (Stanford IRB-38480) retrospective study we reviewed cervical cancer patients treated definitively with chemoradiation and included those who had an additional MR imaging study during the course of their treatment at our institution. Patients were non-pregnant women who were 18 years or older with biopsy proven cervical cancer (squamous or adenocarcinoma). Additionally, patients had no prior history of pelvic radiation or hysterectomy and were classified according to the International Federation of Gynecology and Obstetrics (FIGO) 2018 staging system [29].

Twenty-five cervix cancer patients treated with radiotherapy as part of their standard of care, who underwent FDG PET/CT and IVIM MRI at pre-treatment as well as IVIM MRI at pre-brachytherapy (on-treatment) were retrospectively evaluated. Five of the patients were later excluded from the study due to interruptions during treatment (n = 3) or incomplete imaging datasets (n = 2). Each patient was imaged prior to radiation both using FDG PET/CT to assess metabolic activity and IVIM MRI to assess tumor perfusion and cellularity. All patients underwent concurrent chemotherapy using cisplatin, external beam radiation therapy (EBRT), and high dose rate (HDR) brachytherapy as to definitive treat their cervical cancer, where repeat MRI was performed prior to brachytherapy (3.3 ± 1.9 weeks, 43.3 ± 8.1 Gy). Patients planning tumor volumes were treated using EBRT to 48.6 Gy with an integrated boost to 58.05 Gy to involved lymph nodes as part of their standard of care.

### 2.2 Image acquisition and analysis

Detailed information regarding image acquisition and analysis performed in this study are outlined in the Supplemental Materials section. Briefly, diffusion weighted imaging was acquired at 3 T on a variety of MRIs from multiple vendors (GE Healthcare and Siemens Healthineers) using a free-breathing single shot spin echo planar imaging (EPI) sequence with 11b-values. Additionally, T2-weighted MRI 3D fast spin echo imaging, T1-weighted 3D fast gradient echo MRI, and FDG PET/CT were acquired. Image analysis was performed using MeVisLab (https://www.mevislab.de/) and Matlab R2022b (Mathworks, Natick, Massachusetts, USA). The GTV was contoured by the same radiation oncologist (E.A.K., 15 yrs of experience) and the relative percentage change in gross tumour volume pre- versus on-treatment (ΔGTV) was determined. IVIM MRI maps were generated from the diffusion weighted MR images using a Bayesian fitting of a simplified IVIM model [30]. The mean measurement for each parameter within the GTV (IVIM *D*, IVIM *f*, IVIM D*, and PET SUV) as well as differences between pre- and on-treatment timepoints were determined.

### 2.3 Parametric response mapping

**Fig. 1:**
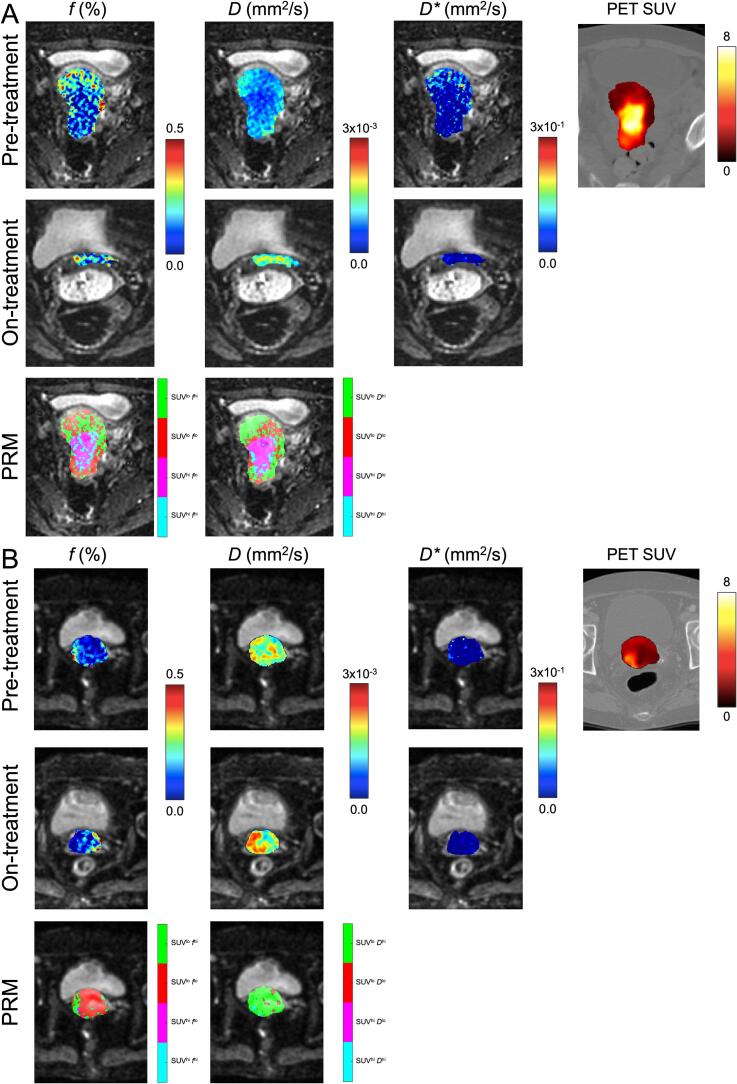
IVIM MRI of two representative cervical cancer patients undergoing CCRT at pre- and on-treatment timepoints as well as and PET imaging at pre-treatment. PRM maps for PET SUV and IVIM *f* as well as PET SUV and IVIM *D* are shown on the bottom row. (A) Patient who responded to treatment by the on-treatment timepoint (age = 41, FIGO Stage IIIC2, histology = SCC, LN Status = Positive). (B) Patient who did not respond to treatment by the on-treatment timepoint (age = 73, FIGO Stage IIB, histology = Adeno, LN Status = Negative).

The generation of parametric response maps was performed first by determining the population means from images acquired prior to treatment to generate thresholds to identify low versus high tumour metabolism (μ_SUV_), low versus high diffused (μ*_D_*) regions, as well as low versus high perfused (μ*_f_*) regions, from PET SUV, IVIM *D* and *f*, respectively. Specifically, the thresholds were generated by calculating the population means (across patients) of the spatially averaged IVIM *D*, IVIM *f*, and PET SUV within the GTVs for each patient. Voxel-wise joint histogram analysis was performed on registered PET and IVIM images at the pre-treatment timepoint to generate labels of co-registered voxels, and then the two population mean thresholds were used to classify the voxels into the following tumor sub-volumes illustrated in Fig. 1: 1) highly metabolic and with low cellular density (SUV^hi^*D*^hi^) in cyan, 2) highly metabolic and with high cellular density (SUV^hi^*D*^lo^) in magenta, 3) metabolically inactive and with high cellular density (SUV^lo^*D*^lo^) in red, or 4) metabolically inactive and with low cellular density (SUV^lo^*D*^hi^) tissue in green. Similar PRM maps were generated using the joint histogram analysis with SUV and *f*. PRM volumes were then normalized to the total tumor volume to produce a relative volume percentage. PRM analysis was performed using an in-house custom-built software developed using MATLAB R2022b (Mathworks, Natick, Massachusetts, USA) available from the authors online (https://github.com/capaldid/PRM).

### 2.4 Statistics

Shapiro-Wilk tests were used to determine the normality of the data, and non-parametric tests were performed for data that were determined to be not normally distributed. Differences between pre- and on-treatment measurements were determined using Wilcoxon tests. Spearman correlation coefficients (*r*) were used to determine the local relationship between PET SUV and IVIM parameters at pre-treatment on a voxel-on-voxel basis. Additionally, global relationships between individual pre-treatment measurements and changes in GTV were determined using Spearman coefficients (*r*). Results were considered significant when the probability of two-tailed type I error (α) was less than 5 % (p < 0.05). Statistical analysis was performed using GraphPad Prism V9.5.1 (GraphPad Software Inc., La Jolla, California, USA).

## 3 Results

**Fig. 2:**
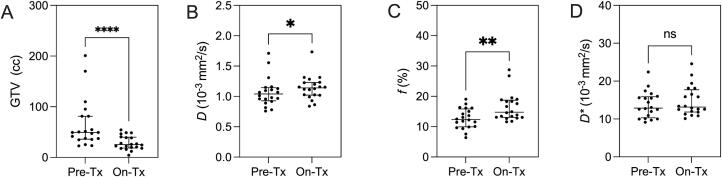
**Pre- vs on-treatment IVIM measurements.** In tumors, on– versus pre- treatment (Tx) tumor volume (A, p < 0.001) significantly decreased, while IVIM *D* (B, p = 0.03) and *f* (C, p = 0.002) significantly increased. IVIM D* did not change (D, p = ns).

Table 1 shows the demographics of the 20 cervical cancer patients included in the study. As illustrated in Fig. 2, GTV significantly decreased (p < 0.001), while both IVIM diffusion (p = 0.03) and perfusion (p = 0.002) significantly increased between on– versus pre- treatment timepoints. Additionally, ΔGTV was not correlated with time between imaging sessions (r = 0.1, p = 0.7).

**Table 1:** Subject Demographic and Imaging Measurements.

**Variables** **mean ± (SD)**	**Pre-treatment (n = 20)**
*Demographics*	
Age, yrs	61 (15)
Time between PET & IVIM, days	14 (17)
Time between 1st & 2nd IVIM, days	61 (13)
*FIGO Stage*	
IIB, n	3
IIIB n	1
IIIC1, n	10
IIIC2, n	4
IVA, n	2
*Histology*	
SCC, n	16
Adeno, n	4
*Lymph Node Status*	
Positive, n	16
Negative, n	4
*Imaging*	
GTV, cc	65 (48)
PET SUV_max_	19 (23)
PET SUV_mean_	3.7 (1.6)
IVIM MRI *D*, 10^-3^ mm^2^/s	1.07 (0.2)
IVIM MRI *f*, %	12.7 (3.3)
IVIM MRI D*, 10^-3^ mm^2^/s	13.7 (3.5)
PRM SUV ^hi^*f*^hi^, %	11 (7)
PRM SUV ^hi^*f*^lo^, %	26 (11)
PRM SUV ^lo^*f*^hi^, %	23 (14)
PRM SUV ^lo^*f*^lo^, %	40 (13)
PRM SUV ^hi^*D*^hi^, %	12 (11)
PRM SUV ^hi^*D*^lo^, %	29 (15)
PRM SUV ^lo^*D*^hi^, %	22 (16)
PRM SUV ^lo^*D*^lo^, %	37 (18)

Note: data are represented as the mean plus or minus standard deviation in brackets. SD: standard deviation; n: sample size; FIGO: International Federation of Gynecology and Obstetrics 2018 staging; SCC: squamous cell carcinoma; Adeno: adenocarcinoma; PET: positron emission tomography; SUV: standard uptake value; IVIM MRI: intravoxel incoherent motion magnetic resonance imaging; *D*: diffusion coefficient; *f*: perfusion fraction; D*: and pseudo-diffusion coefficient; PRM: parametric response mapping; lo: low; hi: high.

Across all patients, voxel-wise correlations between pre-treatment PET SUV and IVIM parameters demonstrated a lack of correspondence of metabolic activity and the diffusion/perfusion patterns (PET SUV vs IVIM *D*: r = -0.15 ± 0.19, and PET SUV vs IVIM *f*: r = -0.08 ± 0.09). Furthermore, we investigated global relationships between pre-treatment PET SUV and IVIM parameters and observed non-significant relationships (PET SUV_mean_ vs IVIM *D*: r = -0.23, p = 0.3, PET SUV_mean_ vs IVIM *f*: r = -0.31, p = 0.2; PET SUV_max_ vs IVIM *D*: r = -0.10, p = 0.7, max PET SUV_max_ vs IVIM *f*: r = -0.13, p = 0.6). These findings suggest complimentary role of PET and DWI IVIM imaging in characterization of cervical tumors.

**Fig. 3:**
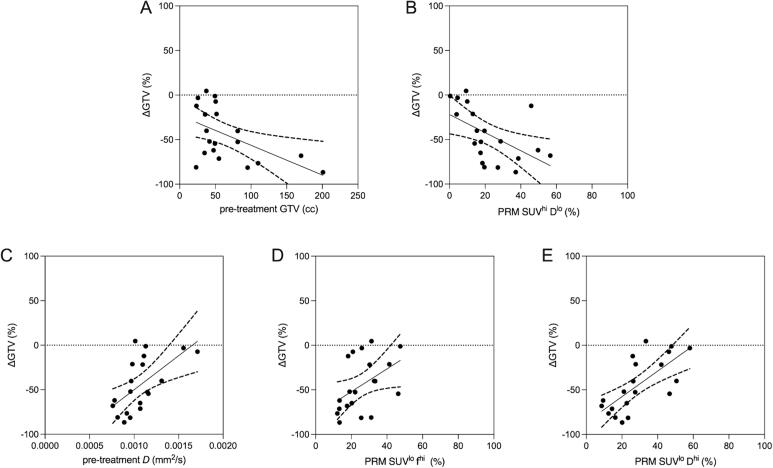
**Relationships between pre-treatment imaging and changes in GTV (pre- vs on-treatment)**. Pre-treatment tumor volume (A, r = -0.45, p = 0.04) and PRM SUV^hi^*D*^lo^ (B, r = -0.65, p = 0.002) regions were negatively related with ΔGTV, while pre-treatment IVIM *D* (C, r = 0.64, p = 0.002), PRM SUV^lo^*f*^hi^ (D, r = 0.52, p = 0.02), and PRM SUV^lo^*D*^hi^ (E, r = 0.74, p < 0.001) regions were positively related with ΔGTV.

**Fig. 4:**
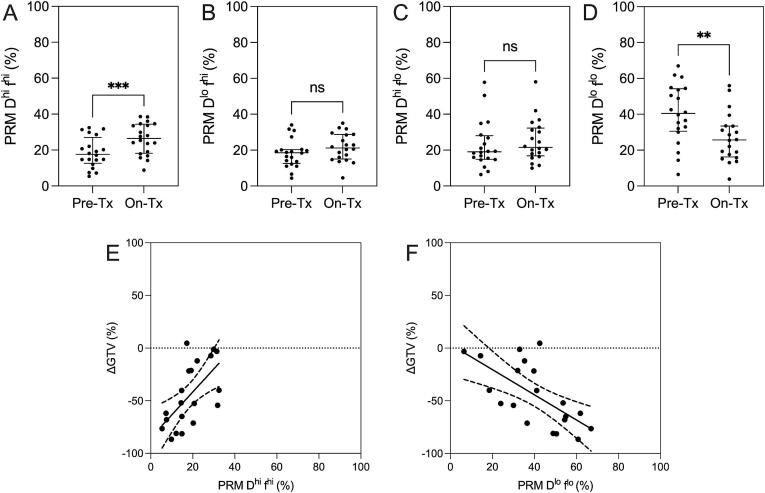
**Parametric response mapping of IVIM *D* and *f*.** In tumors, on– versus pre- treatment (Tx) PRM *D*^hi^*f*^hi^ (A, p < 0.001) significantly increased, while PRM *D*^lo^*f*^lo^ (D, p = 0.001) significantly decreased. PRM *D*^lo^*f*^hi^ (B, p = ns) and PRM *D*^hi^*f*^lo^ (C, p = ns) did not change. Pre-treatment PRM *D*^hi^*f*^hi^ (E, r = 0.62, p = 0.004) was positively related with ΔGTV, while pre-treatment PRM *D*^lo^*f*^lo^ (F, r = -0.64, p = 0.002) was negatively related with ΔGTV.

Table 2 and Fig. 3 both show the relationships between ΔGTV with pre-treatment imaging metrics. GTV_Pre_ (r = -0.45, p = 0.04) and PRM SUV^hi^ *D*^lo^ (r = -0.65, p = 0.002) regions were negatively related with ΔGTV, while pre-treatment IVIM *D* (r = 0.64, p = 0.002), PRM SUV^lo^ *f* ^hi^ (r = 0.52, p = 0.02), and PRM SUV^lo^ *D*^hi^ (r = 0.74, p < 0.001) regions were positively related with ΔGTV. The latter PRM result suggests that larger regions of low cellular density (as represented by elevated *D*) plus low metabolism result in a reduced change in tumor volume on-treatment, potentially representing a radioresistant sub-volume. ΔGTV was neither correlated with ΔD (r = -0.23, p = 0.3) or Δf (r = -0.25, p = 0.3). Furthermore, as IVIM *D* and IVIM *f* were shown to be significant in Fig. 2, we performed PRM mapping of *D* and *f* alone (without PET SUV) and observed significant changes during treatment and relationships with changes in GTV (Fig. 4), potentially suggesting the IVIM alone, without PET, could provide some relevant PRM results albeit their physiologic meaning or mechanism is not as obvious with the PRM SUV vs *f* or *D*. Additionally, the IVIM alone does not provide information regarding metabolic activity which is standardly used for staging, target delineation, and follow-up.

**Table 2:** Univariate Linear Regression Models for Change in Gross Tumor Volume.

**Pre-treatment** **Variables**	**ΔGTV**	
	***r***	**p-value**
**GTV, cc**	**−0.45**	**0.04**
PET SUV_max_	−0.28	0.23
PET SUV_mean_	−0.36	0.12
**IVIM MRI *D*, mm^2^/s**	**0.64**	**0.002**
IVIM MRI *f*, %	0.35	0.13
IVIM MRI D*, mm^2^/s	−0.01	0.95
PRM SUV ^hi^*f*^hi^, %	−0.14	0.56
PRM SUV ^hi^*f*^lo^, %	−0.36	0.12
**PRM SUV ^lo^*f*^hi^, %**	**0.52**	**0.02**
PRM SUV ^lo^*f*^lo^, %	0.08	0.75
PRM SUV ^hi^*D*^hi^, %	0.01	0.96
**PRM SUV ^hi^*D*^lo^, %**	**−0.65**	**0.002**
**PRM SUV ^lo^*D*^hi^, %**	**0.74**	**<0.001**
PRM SUV ^lo^*D*^lo^, %	−0.13	0.57

Correlation coefficients were determined with Spearman (*r*) correlations; Bold values indicate significant relationships (p < 0.05); ΔGTV: relative change in metabolic tumor volume between pre- and on-treatment; PET: positron emission tomography; SUV: standard uptake value; IVIM MRI: intravoxel incoherent motion magnetic resonance imaging; *D*: diffusion coefficient; *f*: perfusion fraction; D*: and pseudo-diffusion coefficient; PRM: parametric response mapping; lo: low; hi: high.

Fig. 1 illustrates two patients who underwent the study with MRI and PET imaging at pre- and on-treatment timepoints, as well as the PRM maps at pre-treatment for both the combined PET SUV plus IVIM perfusion and diffusion. Qualitatively for the patient that was responding to treatment (Fig. 1A), at the pre-treatment timepoint the IVIM perfusion and diffusion was relatively lower in the center of the GTV, as compared to the periphery of the GTV. Alternatively, PET SUV observed the reciprocal trend as compared to the IVIM maps. The visual differences are apparent in the parametric response maps where SUV^hi^ *D*^lo^ and SUV^hi^ *f*^lo^ are more predominant in the core of the GTV, while the reverse is observed in the periphery of the GTV. Additionally, at the on-treatment timepoint, the volume of the GTV was significantly reduced and a redistribution of IVIM perfusion and diffusion was observed within the GTV. Now comparing these observations with the patient that did not respond prior to brachytherapy (Fig. 1B), the pre-treatment timepoint IVIM diffusion was relatively high, and the PET SUV was relatively low in the GTV, compared to the responder. This resulted in the parametric response maps mostly comprising of SUV^lo^ *D*^hi^.

## 4 Discussion

In this study, we investigated the combined role of IVIM MRI and PET in locally advanced cervical carcinoma patients undergoing CCRT as well as utilized parametric response mapping of co-registered PET and IVIM, and we observed the following: 1) significant differences in GTV, IVIM perfusion and IVIM diffusion were observed between pre- and on-treatment timepoints; 2) no relationships between PET SUV and IVIM parameters on a local (voxel-to-voxel) and global (summary statistics) basis; and 3) significant relationships between changes in gross tumor volume and pre-treatment GTV and IVIM diffusion as well as PRM map relative volumes, which leverages information from both IVIM and PET.

First, we observed significant differences in GTV, as well as IVIM perfusion and diffusion between pre- and on-treatment timepoints. These results are consistent with previously published data assessing tumor behavior of cervical cancer patients undergoing CCRT [21]. Specifically, intratumoral perfusion increases while cellularity decreases (i.e. an increase in diffusivity) during treatment, representing changes in the tumor microenvironment. Additionally, IVIM pseudo-diffusion coefficient did not significantly change, which is consistent with previously published work where D* represented the smallest change during treatment as compared to the other IVIM parameters (*D* and *f*) [21].

In this study, IVIM MRI and FDG-PET scans were acquired at the pre-treatment timepoint, and were co-registered within the same frame of reference (i.e., the IVIM MRI – as described in the supplemental section of the manuscript) to facilitate local (voxel-to-voxel) as well as global (summary statistic) comparisons. Both local and global correlations between these two imaging modalities were nonsignificant, producing weak to very weak correlation coefficients. These nonsignificant relationships and spatial mismatch between metabolic activity and cellular perfusion are consistent with previous studies investigating the relationships between FDG-PET and ^15^O perfusion [31] as well as DCE-CT [13]. Additionally, a recent study demonstrated weak or no relationships between PET SUV and IVIM MRI parameters [32]. These weak relationships suggest the potential complementary role and motivating the combined use of PET and IVIM MRI for tumor microenvironment characterization. Consequently, we investigated the utility of PRM [14], [15], [16], [17] to combine the co-registered PET SUV and IVIM MRI, similar to previous work [18], to identify sub-volumes in cervical cancer patients that may predict treatment response to CCRT.

Significant correlations between ΔGTV with pre-treatment imaging parameters were observed − specifically, gross tumor volume, IVIM MRI diffusion, as well as PRM sub-volumes SUV^hi^ *D*^lo^, SUV^lo^ *f* ^hi^, and SUV^lo^ *D*^hi^. Previous work has explored the use of PRM volumes generated from PET SUV and DCE CT blood flow (BF) which demonstrated significant relations with changes in the metabolic tumor volume (MTV – generated from PET imaging), specifically the PRM SUV^lo^ BF^lo^ subvolume [18]. As compared to this previous study, the perfusion volume contribution in the PRM analysis differed: we observed significant relations with PRM SUV^lo^ *f* ^hi^. Additionally, the strongest relationship with changes in GTV was with the PRM SUV^lo^ *D*^hi^, showing a significant positive correlation coefficient. Hence, tumors consisting mostly of PRM SUV^lo^ *D*^hi^ (i.e. low metabolic activity and low cellularity) at pre-treatment, will most likely not change in size during treatment. Alternatively, tumors consisting mostly of PRM SUV^hi^ *D*^lo^ (i.e. high metabolic activity and high cellularity) at pre-treatment, will experience larger changes in tumor volume (as shown in Fig. 3B). We believe that the PRM SUV^hi^ *D*^lo^ sub-volume represents tumor cells that are sensitive to treatment affect (changes in size) as these GTV regions mostly comprised of densely packed (low diffusion/high cellularity) and rapidly proliferating, highly metabolic (high SUV) cells. Based on the presented results, the identified sub-volumes could have the potential to predict treatment-response.

There are some limitations in this current study. First, we had a relatively small sample size, potentially underpowering our presented results. Due to loss to follow up, this study did not include the component of standard PET-CT response assessment at 3 months post-therapy. In addition to the relatively low number of patients in this study, the utility of the PRMs as predictors of post-therapy response needs to be tested in a larger, prospective study. Additionally, this study did not include a component of long-term follow up where the true treatment success or failure was evaluated (such as changes in the metabolically active tumour volume), thus the results observed here certainly warrant caution. Next, the average time between the pre-treatment IVIM MRI scan and the initial PET scan was 13.8 ± 16.6 days, resulting in the need for image registration between these two image sets. We acquired DWI MRI on multiple MRI vendors (GE and Siemens) and did not explicitly evaluate the impact of IVIM due to vendor type, which could introduce variability in IVIM measurements, albeit the values calculated were similar to those previously publish in cervical cancer [33]. In this study, we used a Bayesian-fitting simplified two-compartment model to extract the IVIM parameters from DWI MRI, which have been shown to outperform both linear and nonlinear least squares fitting in terms of coefficients of variation (repeatability measurements) [34], [35]. Image registration poses a challenge in PRM as this method relies on a voxel-wise analysis. Previous studies have reported the effects of image registration errors on tissue misclassification for PRM in the liver and the lungs [14], [36]. Additionally, the gold fiducials placed around the tumor may also impact the IVIM metrics, albeit not explicitly evaluated in this study. It should be noted that the lack of voxel-wise correspondence of metabolic activity and the diffusion/perfusion patterns could be to a certain degree result of registration error (as rigid body registration was employed) as well as distortion in the EPI sequence used this this study. This is why we also evaluated population-based correlations between PET and IVIM spatially averaged values which would not be affected by registration errors. We must also acknowledge that there were differences in the total dose received at the on-treatment timepoint (prior to brachytherapy) at the time of the second IVIM MR imaging session potentially resulting in heterogeneity in the overall IVIM measurements, albeit none of the IVIM measurements at the on-treatment timepoint were correlated with dose suggesting there was little effect. While traditional measurements of RECIST have been shown to relate to treatment response [37], volume-based measurements were used in this study for convenience as baseline volumes were available at baseline as part of the clinical planning process and radiation oncologist could easily contour the volumes on the on-treatment images. Previous work has shown mid-treatment changes in tumor volume was a predictor of local control rate in cervical cancer patients receiving CCRT [27], [28]. Future work will investigate exploring multi-PRM mapping [38] to combine all three metrics (PET SUV, IVIM *D*, and IVIM *f*) into a combined space in a larger sample size.

In summary, IVIM MRI and PET imaging was performed on patients with locally advanced cervical carcinoma undergoing CCRT and we observed that both IVIM perfusion fraction and diffusion coefficient increased during treatment – reflecting changes in the tumor microenvironment. Weak relationships between PET and IVIM were observed, suggesting the potential complementary role of the combined use of these modalities for tumor characterization. Lastly, PRM, generated from PET and IVIM MRI, was applied and sub-volumes were identified which may predict treatment response. The complementary information provided from PET and IVIM, combined using PRM, may assist in decision-making to individualize therapies.

## CRediT authorship contribution statement

**Dante P.I. Capaldi:** Conceptualization, Methodology, Software, Validation, Formal analysis, Investigation, Data curation, Writing – original draft, Writing – review & editing, Visualization. **Jen-Yeu Wang:** Conceptualization, Methodology, Software, Validation, Formal analysis, Investigation, Data curation, Writing – review & editing, Visualization. **Lianli Liu:** Methodology, Investigation, Data curation, Writing – review & editing. **Vipul R. Sheth:** Conceptualization, Methodology, Investigation, Data curation, Writing – review & editing. **Elizabeth A. Kidd:** Conceptualization, Methodology, Investigation, Data curation, Writing – review & editing, Supervision, Resources, Project administration. **Dimitre H. Hristov:** Conceptualization, Methodology, Software, Validation, Formal analysis, Investigation, Data curation, Writing – original draft, Writing – review & editing, Visualization, Supervision, Resources, Project administration.

## Declaration of Competing Interest

The authors declare that they have no known competing financial interests or personal relationships that could have appeared to influence the work reported in this paper.
