# Supplementary material for: Parametric response mapping of co-registered intravoxel incoherent motion magnetic resonance imaging and positron emission tomography in locally advanced cervical cancer undergoing concurrent chemoradiation therapy
Source: Phys Imaging Radiat Oncol. 2024 Aug 18;31:100630. doi: 10.1016/j.phro.2024.100630 (PMC11387531; doi:10.1016/j.phro.2024.100630)
Supplement: Supplementary Data 1 [file mmc1.docx]

**A1. SUPPLEMENTAL MATERIALS**

Diffusion weighted imaging was acquired at 3T on a variety of MRIs from multiple vendors (GE Healthcare and Siemens Healthineers) using a free-breathing single shot spin echo planar imaging (EPI) sequence with 11 b-values of 0, 10, 20, 30, 50, 80, 100, 150, 300, 500, 800 s/mm^2^ (TR = 11000 ms; TE = 86 ms; number-of-averages = 1, 1, 1, 1, 1, 1, 1, 1, 1, 1, 8; flip-angle = 90 deg; slice thickness = 3.0 mm) – protocols configured by a Radiologist (V.R.S.). Additionally, T2-weighted 3D fast spin echo imaging (GE: TR = 2002 ms, TE = 99.3 ms, flip-angle = 90 deg, slice thickness = 1.2 mm; Siemens: TR = 1700 ms, TE = 105 ms, flip-angle = 160 deg, slice thickness = 1.2 mm) and T1-weighted 3D fast gradient echo imaging were acquired for GTV contouring (GE: TR = 4.6 ms, TE = 1.03 ms, flip-angle = 15 deg, slice thickness = 1.4 mm; Siemens: TR = 3.71 ms, TE = 1.75 ms, flip-angle = 9 deg, slice thickness = 1.5 mm). Image analysis was performed using MeVisLab ([www.mevislab.de/](http://www.mevislab.de/)) and Matlab R2022b (Mathworks, Natick, USA). FDG PET/CT scans were reconstructed, and attenuation corrected using Siemens True Time-of-Flight (ultraHD-PET) algorithm with 2 iterations and 21 subsets (size = 4.073 x 4.073 x 3 mm^3^, filter=none). PET images were converted into standardized uptake value (SUV) based on injected activity and patient body weight.

The GTV was contoured by the same radiation oncologist (E.A.K., 15 yrs of experience) on the T2 weighted sequence in ARIA oncology system’s contouring application (Varian Medical Systems, Palo Alto, California, USA) with reference to the post contrast imaging and planning CT and PET scans. The planning CT and PET/CT scans, one set of pre-EBRT MRI (pre-treatment) sequences and GTV contour, and one set of pre-brachytherapy MRI (on-treatment) sequences and GTV contour were exported out of the treatment planning system and/or picture archiving and communication system in the Digital Imaging and Communications in Medicine (DICOM) format. The sequences were registered together to facilitate an accurate conversion of each GTV contour into a mask on the IVIM sequence that was subsequently reviewed by the radiation oncologist. The registration steps performed to map images to the IVIM MRI space are outlined below, where the final voxel size of the PRM maps were 1.923 x 1.923 x 3mm^3^ and resampled using the bicubic interpolation function. The relative percentage change in gross tumour volume pre- versus on-treatment (ΔGTV) was determined using the following equation:

|  | $\Delta GTV= \frac{\left( {GTV}_{On} \left[ \mathrm{mL} \right]-{GTV}_{Pre}\left[ \mathrm{mL} \right] \right)}{{GTV}_{Pre}\left[ \mathrm{mL} \right]}\times100$ | (A.1) |
| --- | --- | --- |

where GTV_Pre_ and GTV_On_ are the pre- and on-treatment GTVs, respectively.

IVIM MRI maps were generated from the diffusion weighted MR images using a Bayesian fitting of a simplified IVIM model [1]. Specifically, IVIM maps were generated based on the two-compartment model [2]:

|  | $S\left( b \right)=S_{0}(\left( 1-f \right)e^{-bD}+fe^{-bD^{*}})$ | (A.2) |
| --- | --- | --- |

where $S\left( b \right)$is the signal at a diffusion weighted image (based on b-value), $S_{0}$ is the signal without diffusion weighting, $D$ is the diffusion coefficient, $f$ is the perfusion fraction, and $D^{*}$ is the pseudo-diffusion coefficient. All eleven b-values (11 b-values of 0, 10, 20, 30, 50, 80, 100, 150, 300, 500, 800 s/mm^2^) we used in the fitting. The mean measurement for each parameter within the GTV (IVIM *D*, IVIM *f*, IVIM *D**, and PET SUV) as well as differences between pre- and on-treatment timepoints were determined.

In this study, multiple registration steps were performed as part of the clinical workflow for each of these patients where the primary Radiation Oncologist (E.A.K.) was involved in all the registration steps for defining treatment volumes. First, the PET/CT is rigidly registered to the planning CT in Eclipse (Varian Medical Systems, Palo Alto, California, USA), where these images are acquired back-to-back as part of their simulation, and registration is mostly to account for any patient movement during the imaging session. Next, the T2 MRI were also registered to the planning CT in Eclipse (Varian Medical Systems, Palo Alto, California, USA), where the GTV was defined with the aid of the fused MRI, which was registered to the planning CT for target definition. This registration step (saved as a DICOM registration file) was reversed for the purpose of mapping the PET/CT to the IVIM MRI in the same space. All these DICOM files were exported out of the treatment planning system and read into custom MeVisLab scripts. Lastly, the T2 MRI was registered to the IVIM MRI (both acquired during the same MR imaging session) using a normalize mutual information algorithm in the MeVisLab script to correct for potential small intra-imaging session motion. In this last step, the primary Radiation Oncologist (E.A.K.) reviewed all registrations and contours transferred to the IVIM MRI. In patients where gold fiducial markers were implanted around the tumor, the primary Radiation Oncologist also used these markers in verifying the registration. An example of the final registration is illustrated in Figure S1.

**
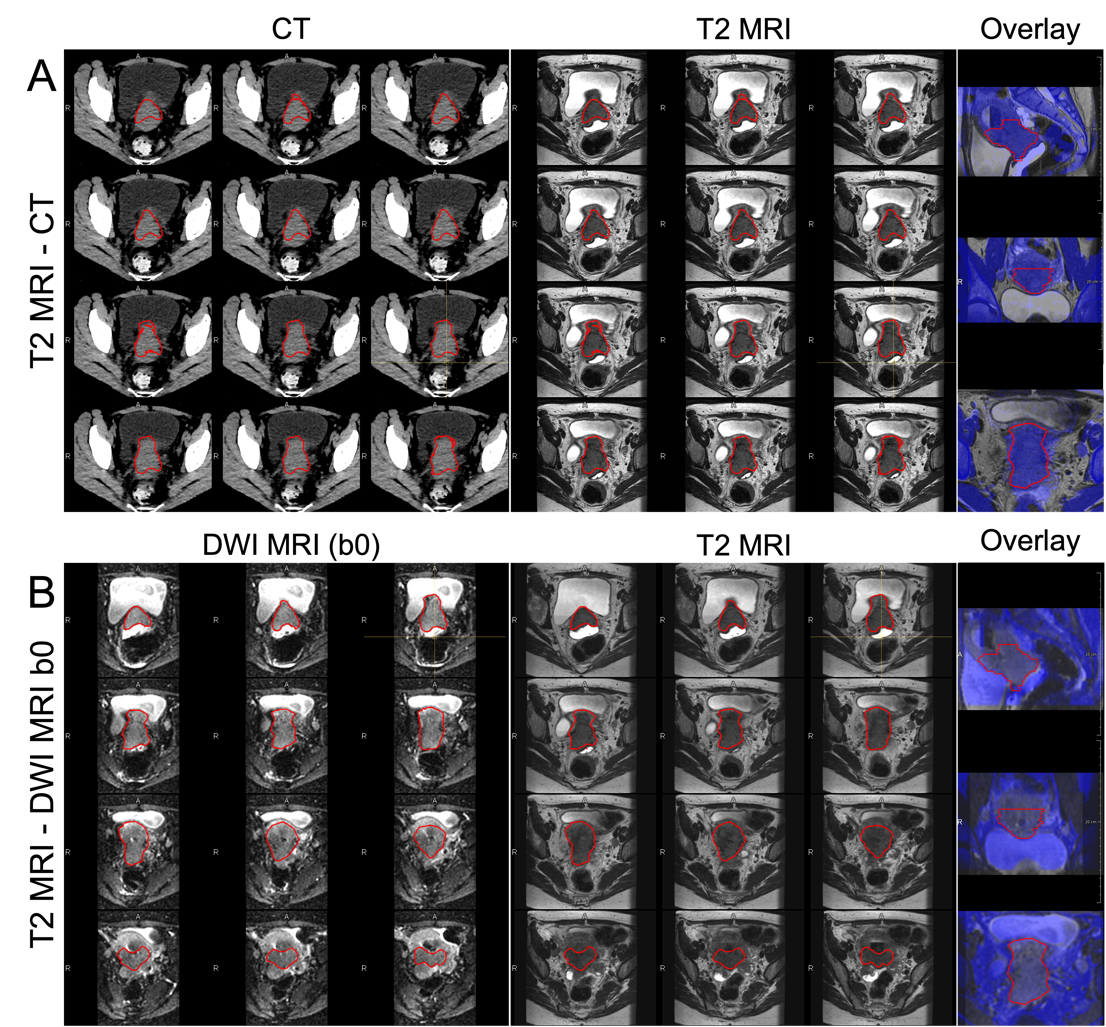
**

**Figure S1.** Example image registration between T2 MRI with (A) CT (from PET / CT) and (B) DWI MRI (b0 image). The overlay images represent T2 MRI in grayscale with either CT or DWI MRI in blue. The red contour represents the GTV.

**A.2. REFERENCES**

[1] Jalnefjord O, Andersson M, Montelius M, Starck G, Elf A-K, Johanson V, et al. Comparison of methods for estimation of the intravoxel incoherent motion (IVIM) diffusion coefficient (D) and perfusion fraction (f). MAGMA 2018;31:715–23. https://doi.org/10.1007/s10334-018-0697-5.

[2] Le Bihan D, Breton E, Lallemand D, Aubin ML, Vignaud J, Laval-Jeantet M. Separation of diffusion and perfusion in intravoxel incoherent motion MR imaging. Radiology 1988;168:497–505. https://doi.org/10.1148/radiology.168.2.3393671.
